# Intra-articular injection of recombinant TRAIL induces synovial apoptosis and reduces inflammation in a rabbit knee model of arthritis

**DOI:** 10.1186/ar1867

**Published:** 2005-12-16

**Authors:** Qingping Yao, Dai-Wu Seol, Zhibao Mi, Paul D Robbins

**Affiliations:** 1 Department of Molecular Genetics and Biochemistry, 200 Lothrop Street, University of Pittsburgh School of Medicine, Pittsburgh, PA 15261, USA; 2 Department of Surgery, 200 Lothrop Street, University of Pittsburgh School of Medicine, Pittsburgh, PA 15261, USA

## Abstract

We demonstrated previously that local, intra-articular injection of an adenoviral vector expressing human tumor necrosis factor-related apoptosis-inducing ligand (TRAIL) in a rabbit knee model of inflammatory arthritis stimulated synovial apoptosis and reduced inflammation. To examine whether intra-articular injection of recombinant chimeric human TRAIL protein (rTRAIL) also induces apoptosis of proliferating rabbit synovium and reduces inflammation, we used an experimental rabbit arthritis model of rheumatoid arthritis, induced by intra-articular introduction of allogeneic fibroblasts genetically engineered to secrete human IL-1β. Analysis of synovium isolated from the rabbits treated with intra-articular injection of rTRAIL, relative to saline control, showed areas of extensive acellular debris and large fibrous regions devoid of intact cells, similar to adenoviral mediated TRAIL gene transfer. Extensive apoptosis of the synovial lining was demonstrated using TUNEL analysis of the sections, corresponding to the microscopic findings in hematoxylin and eosin staining. In addition, leukocyte infiltration into the synovial fluid of the inflamed knee joints following rTRAIL treatment was reduced more than 50% compared with the saline control. Analysis of the glycosaminoglycan synthetic rate by cultured cartilage using radiolabeled sulfur and cartilage histology demonstrated that rTRAIL did not adversely affect cartilage metabolism and structure. Analysis of serum alanine aminotransferase showed that intra-articular injection of rTRAIL did not have adverse effects on hepatic function. These results demonstrate that intra-articular injection of rTRAIL could be therapeutic for treating pathologies associated with rheumatoid arthritis.

## Introduction

Tumor necrosis factor (TNF)-related apoptosis-inducing ligand (TRAIL) is a type II transmembrane protein that was initially identified according to the homology of its extracellular domain with CD95L (FasL), TNF-α and lymphotoxin-α [1,2]. TRAIL induces apoptosis by binding and cross-linking the death-domain containing receptors, TRAIL-R1 (DR4) and TRAIL-R2 (DR5) [3]. Other TRAIL receptors such as TRAIL-R3 and TRAIL-R4 [4] act as decoy receptors that are able to inhibit the cytotoxic effects of TRAIL. Interestingly, TRAIL is able to induce apoptosis of a wide variety of human tumor cells, but generally appears not to affect normal cells [5]. Thus, systemic administration of recombinant TRAIL protein (rTRAIL) is being developed clinically for the treatment of cancer.

Rheumatoid arthritis (RA) is a debilitating systemic autoimmune disease characterized by chronic inflammation of distal diarthrodial joints. Affected joints exhibit inflammatory cell infiltration and synovial hyperplasia that contribute to the progressive degradation of cartilage and bone [6,7]. The removal of the synovial pannus by either surgery [8] or radioactive isotopes [9] has proven to be useful in treating RA in certain cases, resulting in pain relief and better outcome. These methodologies, however, have inherent limitations in the treatment of multiple diseased joints. Thus, direct intra-articular injection of agents such as recombinant proteins able to induce synovial apoptosis may offer a safe therapeutic approach to remove the synovial pannus.

Previously, we have shown that adenoviral mediated gene transfer of p53 [10] and FasL [11] to inflamed rabbit knee joints results in induction of significant synovial apoptosis as well as reduction of the extent of leukocytic infiltration. More recently, we demonstrated that adenoviral mediated gene transfer of membrane bound human TRAIL [12] induced apoptosis of both rabbit and human synovial cells in culture, albeit only at a high multiplicity of adenoviral infection. In addition, intra-articular injection of the adenoviral (Ad)-TRAIL vector resulted in extensive apoptosis, similar to that observed with FasL, but was able to reduce joint inflammation in contrast to the inflammatory effect of FasL gene transfer. These gene transfer studies suggest that expression of certain apoptotic agents intra-articularly could be therapeutic to treat certain pathologies associated with RA. Currently, no viral or non-viral vectors are suitable for efficient and safe intra-articular gene transfer, however, especially if there is a need for repeat dosing.

The ability of rTRAIL to induce tumor specific apoptosis as well as the ability of intra-articular TRAIL gene transfer to induce synovial apoptosis suggest that intra-articular injection of rTRAIL also might be able to induce apoptosis of hyperplastic synovium. In this report, we have examined the ability of rTRAIL to induce synovial apoptosis *in vivo *in inflamed rabbit knee joints following intra-articular injection. Similar to the effects of intra-articular injection of Ad-TRAIL, injection of exogenous rTRAIL was able to induce synovial apoptosis in arthritic joints of rabbits as well as reduce inflammation. In addition, there was no adverse effect observed locally on cartilage metabolism or systemically on hepatic function. These results suggest that local injection of rTRAIL could be therapeutic for treating pathologies associated with RA.

## Materials and methods

### Preparation and culture of synovial fibroblasts

Synovial tissues from rabbits with IL-1β induced arthritis were minced and digested with 0.2% collagenase type I (Clostridiopeptidase, Sigma, St. Louis, MO, USA). A recovered single cell suspension after washing three times was cultured in 10% fetal bovine serum Dulbecco's modified Eagle's medium in T25 cm^2 ^flasks in a humidified incubator supplied with 5% CO_2 _at 37°C. The synovial fibroblasts obtained after passage of primary synovial cells in culture three times were used in the described experiments.

### MTT assay of the viability of synoviocytes following TRAIL protein treatment

A human TRAIL cDNA fragment (corresponding to amino acids 114 to 281) obtained by PCR was cloned into the pET-23d (Novagen, Madison, WI, USA) plasmid, and expressed protein was purified using the His-band Resin and Buffer Kit (Novagen) [13]. Analysis of the purified rTRAIL protein demonstrated that both trimeric and dimeric TRAIL were present at a ratio of 4:1 [13] (data not shown). This rTRAIL was used for both *in vitro *and *in vivo *experiments. To test the effect of rTRAIL on cell proliferation in cell culture, 1 × 10^5 ^synovial fibroblasts per well of rabbit were plated and grown to near confluence in 24-well culture plates. The cells in culture were then co-incubated with various doses of rTRAIL for 48 h and the cell viability of each well was determined using a 3-(4,5-dimethylthiazol-2-yl)-2,5-diphenyltetrazolium bromide (MTT) assay [14]. The value of saline control was assigned 100%, and the number of rTRAIL treated cells was relative to control.

### Establishment of the arthritis model

To induce arthritis of rabbit with IL-1β, we used IL-1-producing cells (IL-1 cells). Allogeneic rabbit synovial fibroblasts were engineered to express human IL-1β by transduction with the retroviral vector DFG-human IL-1-neo, which contains the cDNAs for hIL-1β and neomycin phosphotransferase (neor), [15] and cultured in 75 cm^2 ^flasks in the presence of G418 at a concentration of 0.5 mg/ml. The secreted IL-1 level in the supernatant of the maintained cell culture was measured regularly by ELISA and was >150 ng hIL-1β/10^6 ^cells/48 h. After the IL-1 cells in culture were trypsinized, resuspended and washed in saline three times, 5 × 10^5 ^cells in a total volume of 0.25 ml saline in 1 ml syringe were implanted in naive knee joints via injection through the patellar tendons of New Zealand white female rabbits weighing 5 to 6 pounds each. The rabbits generally developed arthritis within 24 h following intra-articular injection of IL-1 cells. All of the rabbit experiments were approved by the University of Pittsburgh Institutional Animal Care and Use Committee (IACUC).

### Intra-articular injection of rTRAIL and collection of tissues

To test the potential *in vivo *biological efficacy of rTRAIL, 5 and 20 μg of rTRAIL, was injected intra-articularly into both knees of two rabbits on day three following the implantation of IL-1β cells; saline was injected into the knees of two other animals as controls. At 48 h post injection, the lavage synovial fluid (LSF), synovium, and cartilage were taken from each individual knee joint of euthanized rabbits in all three groups for counting of white blood cells, histopathological inspection, TUNEL (terminal deoxynucleotidyl transferase-mediated dUTP nick end labeling) staining, and determination of cartilage metabolism.

### Leukocyte count

To determine if rTRAIL affects joint inflammation, LSF was obtained from each knee joint of the rabbits before and after injection of rTRAIL. EDTA anti-coagulated LSF was cleared of red blood cells with lysis buffer and counted with a hematocytometer for white blood cells; results are expressed as ×10^6 ^cells per ml.

### Histopathological inspection of synovium

To observe the possible *in vivo *killing effect of rTRAIL on synovial linings, synovial tissues of knees were taken at 48 h post injection and individually fixed immediately in 10% buffered formalin in a 50 ml polypropylene cornical tube. After fixation for 5 to 10 days, the synovium was processed, paraffin-embedded, sectioned at 5 um, and hematoxylin and eosin (H&E) stained. Three stained sections made from each synovium sample were prepared. Two experienced investigators blindly inspected each individual section for cell death and grading based upon the magnitude of areas with dead cells and debris residing in the synovium on a scale from 0 to 4.

### TUNEL staining of synovium for apoptosis

**Figure 1:**
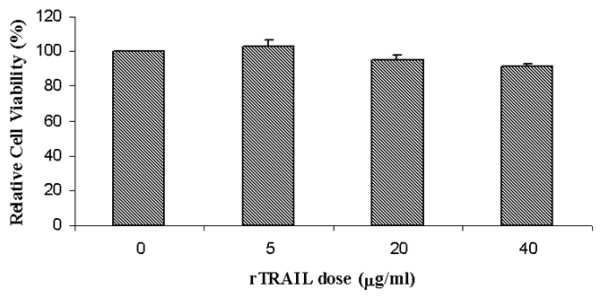
Recombinant TRAIL induces apoptosis and reduces white blood cell infiltrate in arthritic rabbit knees. **(a) **Recombinant TRAIL (rTRAIL) results in massive death of synovial cells of arthritic rabbit knees. Arthritic rabbit knee joints, induced by intra-articular injection of IL-1β producing cells, were then treated intra-articularly with 5 and 20 μg of rTRAIL per knee or saline. Sections of the synovium from the 20 μg of rTRAIL treated rabbits were analyzed at 48 h post-injection by hematoxylin and eosin ((H&E; 100×) and TUNEL (100×) staining. Examples of apoptotic cells or regions of apoptosis are indicated by the arrows. **(b) **Intra-articular injection of rTRAIL reduces joint inflammation. Lavage synovial fluid (LSF) from saline and rTRAIL treated arthritic rabbit knee joints were aspirated 48 h following intra-articular injection and leukocytes counted using a hemacytometer. The data were expressed as mean ± standard error of the mean ×10^6 ^per ml LSF. WBC, white blood cell.

To confirm if the death of synovial cells found in H&E slides was apoptotic cell death, three blank sections corresponding to each H&E stained synovium slide were deparaffined and the synovium stained for apoptosis using TdT-FragELTM DNA Fragmentation Detection Kit (Oncogene, Cambridge, MA, USA). The apoptotic events present on sections were evaluated on a scale of 0 to 4 with regard to presence or absence of apoptotic events as well as the scope of apoptotic regions. A representative sample section from either treatment or saline group is shown (Figure 1a).

### Determination of glycosaminoglycan synthesis in cartilage

To assess the possible impact of rTRAIL on the articular cartilage, small pieces of articular cartilage were shaved from the femoral condyles of rabbit knees and weighed individually. Approximately 20 mg of cartilage obtained from each joint was then incubated in 1 ml of serum-free medium with 40 μCi of ^35^SO4^-2 ^at 37°C for 24 h. The culture medium was harvested and stored at -20°C, and glycosaminoglycans (GAGs) were extracted from the cartilage shavings remaining in the wells by incubation in 0.5 ml of 0.5 M NaOH at 4°C for 24 h, with gentle shaking on a shaker. Following chromatographic separation of free and incorporated ^35^SO4^-2 ^using PD-10 columns (Pharmacia, Piscataway, NJ, USA), radiolabeled GAGs released into the culture medium and recovered by alkaline extraction were quantified using scintillation counting. The value of saline control joints was assigned 100% and the results of the rTRAIL treated joints shown relative to the saline control.

### Comparison of rTRAIL and membrane bound Ad-TRAIL treatment efficacy

To compare the efficacy of rTRAIL in the treatment of rabbit joint synovitis with that of Ad-mediated membrane bound murine TRAIL (Ad-mTRAIL) gene transfer, rabbit arthritic knee joints that were induced by intra-articular injection of IL-1β cells were then treated intra-articularly with 5 or 20 μg of rTRAIL per knee or saline and 1 × 10^11 ^particles of Ad-mTRAIL vectors or adenoviral Ad-Lac Z vectors for 48 h. [12]. The rabbits were sacrificed and joint capsules were sectioned to evaluate severity of synovitis by H&E and TUNEL staining.

### Serum alanine aminotransferase test

To assess the systemic toxicity of TRAIL protein, the rabbits were bled for sera to test for hepatic function and cellular injury. Serum alanine aminotransferase levels were measured using the Opera Clinical Chemistry System (Bayer Co, Tarrytown, NY, USA).

## Results

### rTRAIL induces synovial apoptosis following intra-articular injection

**Figure 2:**
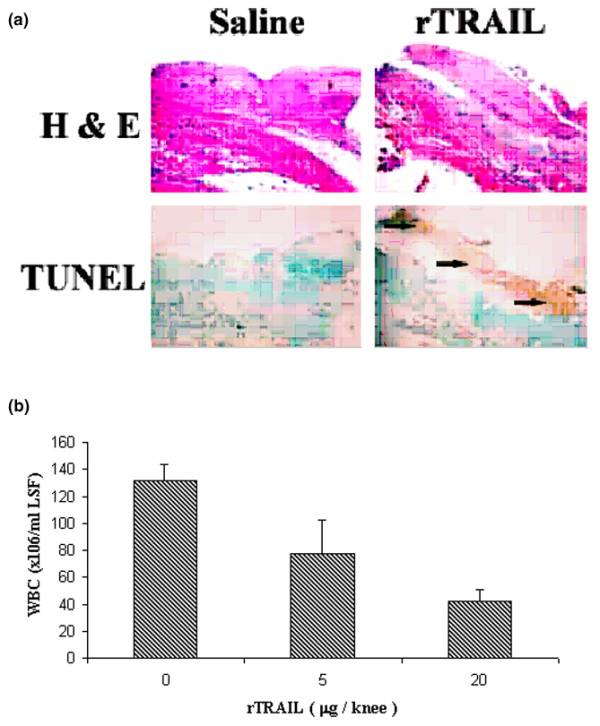
Recombinant TRAIL (rTRAIL) showed no significant inhibition of synoviocytes of rabbit. Primary synoviocytes prepared from arthritic knee joints by digesting the synovium with collagenase of rabbit were cultured and the primary synovial fibroblasts were acquired after passage in culture three times. The cells were cultured and grown to confluent in 24-well plates overnight and then co-incubated in duplicate with 5, 20, and 40 μg of rTRAIL for 48 h. The cell viability was measured using a MTT assay and the data expressed as percentage viability in rTRAIL treated cells relative to non-treated cells.

To determine whether rTRAIL can induce apoptosis of rabbit synoviocytes, synovial fibroblasts from inflamed knee joints of rabbit were cultured in 24-well culture plates and then co-incubated with various doses (5, 20, and 40 μg/ml) of rTRAIL for 48 h. rTRAIL exhibited a small but insignificant effect on rabbit synovial fibroblast viability relative to saline control as determined using an MTT assay (Figure 2). Similarly, addition of increasing doses of rTRAIL to two different cultures of human RA synovial fibroblasts showed marginal effects on cell viability (data not shown). These results suggest that rTRAIL is not effective in inducing apoptosis of synovial cells in culture. Our previous studies with adenoviral gene transfer of membrane bound TRAIL, however, showed that it too was relatively inefficient in inducing synovial apoptosis, requiring a high multiplicity of infection to confer apoptosis in culture. Thus it appears as if TRAIL, either administered as a recombinant protein or by adenoviral gene transfer, is relatively ineffective in inducing apoptosis of both rabbit and human synovial fibroblasts in culture.

In contrast to the cell culture results using human and rabbit synovial fibroblasts, we demonstrated previously that intra-articular adenoviral mediated gene transfer of TRAIL into inflamed rabbit knees *in vivo *resulted in extensive apoptosis, suggesting that the proliferating synoviocytes *in vivo *are more susceptible to TRAIL than cultured synovial fibroblasts. Therefore, we have examined the ability of intra-articular injection of rTRAIL to induce apoptosis of synovium *in vivo *in inflamed rabbit knees. At 48 h post injection of rTRAIL into inflamed rabbit knees, synovial tissue from each knee joint was isolated and fixed. Histological examination of H&E stained synovium showed regions devoid of intact cells, suggesting extensive cell death throughout the synovium within 48 h following intra-articular injection of rTRAIL (Figure 1a). Analysis of the synovial cell sections by TUNEL staining clearly showed that there is extensive apoptosis following rTRAIL injection. Analysis of the extent of apoptosis showed a marked increase in apoptosis in rTRAIL treated joints relative to control joints (Table 1). The extent of apoptosis observed was similar to that observed following adenoviral gene transfer of TRAIL and FasL.

### rTRAIL reduces joint inflammation

As TRAIL has been shown to induce apoptosis of activated T cells [16,17], we also examined if the intra-articular injection of rTRAIL might also be effective in reducing the extent of the white blood cell infiltrate. For this purpose, the number of leukocytes in the synovial fluid that was aspirated from arthritic knee joints of rabbits was monitored before and after injection of rTRAIL. Leukocytic infiltration into the joints of rTRAIL treated animals was reduced greater than 50% compared to the saline control (Figure 1b), suggesting that rTRAIL can inhibit the leukocytic infiltrate into the joint space or can induce apoptosis of the infiltrating cells.

### rTRAIL treatment does not affect cartilage or confer systemic toxicity

**Figure 3:**
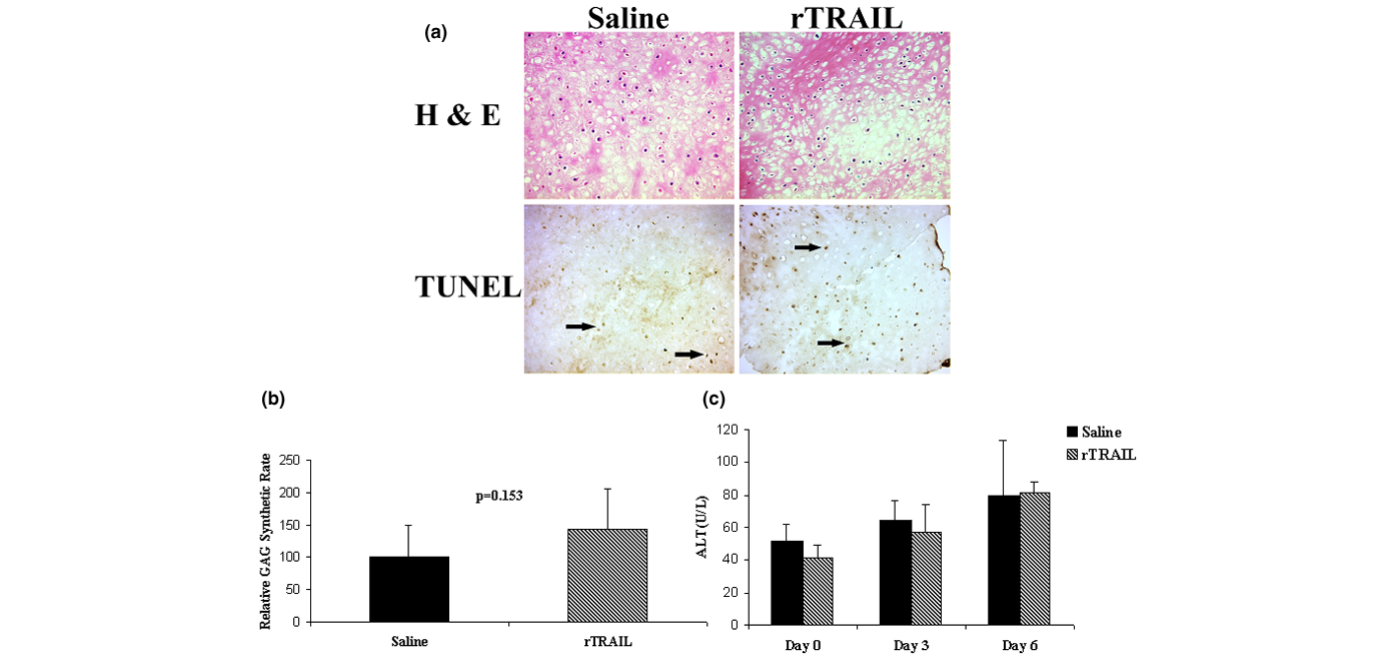
Analysis of cartilage and systemic toxicity of recombinant TRAIL (rTRAIL). **(a) **To assess the toxicity of TRAIL protein on joints, the shaved cartilage under synovial lining was sectioned for evaluation of cartilage structure. Histological examination of hematoxylin and eosin (H&E) and TUNEL stained cartilage sections (100×) made from rTRAIL treated and saline control joints are shown. Examples of apoptotic cells are indicated by the arrows. **(b) **Analysis of glycosaminoglycan (GAG) synthesis by cartilage following rTRAIL injection. Cartilage shavings were taken from the femoral condyles of rabbits 48 h post-injection and analyzed for GAG synthesis as described in Materials and methods. The results are shown as percentage change (mean ± standard error of the mean) following rTRAIL treatment relative to 100% in the saline control. **(c) **Analysis of possible systemic adverse effects caused by intra-articular injection of rTRAIL. The effect of rTRAIL injection on hepatic function and injury was examined by measuring serum alanine aminotransferase (ALT) levels in rabbits prior to injection of IL-1β expressing cells (day 0), 3 days post-injection of IL-1β expressing cells (day 3) and 3 days post-injection of either saline or rTRAIL into arthritic rabbit knees (day 6).

Although induction of synovial apoptosis would be therapeutic, clearly induction of chondrocyte death or dysfunction locally or systemic toxicity would be disadvantageous. Thus we examined whether rTRAIL also affects cartilage metabolism and structure as well as hepatic function as a marker for systemic effects while inducing apoptosis of the synovial lining. To evaluate cartilage structure damage, the shaved cartilage under synovial lining was sectioned for histological analysis and TUNEL staining. Histological examination of H&E stained cartilage sections from rTRAIL treated and saline control joints showed cartilage destruction due to IL-1β caused inflammation and evidence of apoptosis (Figure 3a). There was no evidence, however, of additional apoptosis caused by rTRAIL in cartilage as determined by H&E as well as by TUNEL staining. The effect of rTRAIL on cartilage metabolism also was evaluated. The cartilage was shaved from arthritic knee joints receiving protein or saline, cultured and pulsed with ^35^SO4^-2 ^for analysis of GAG synthesis as an indicator of cartilage metabolism. No significant differences in GAG synthesis were observed in the cultured cartilage shavings between the rTRAIL-treated joints and control (Figure 3b). These data suggest that the intra-articular injection of exogenous rTRAIL, able to induce apoptosis of synovium, appears not to affect articular cartilage. Furthermore, systemic toxicity of rTRAIL administration was examined by measuring serum alanine aminotransferase levels, which increase due to hepatic injury. There was no additional toxicity due to rTRAIL treatment (Figure 3c), suggesting that local delivery of rTRAIL did not confer systemic adverse effects.

### rTRAIL has similar apoptotic effects as adenoviral mediated TRAIL gene transfer

**Figure 4:**
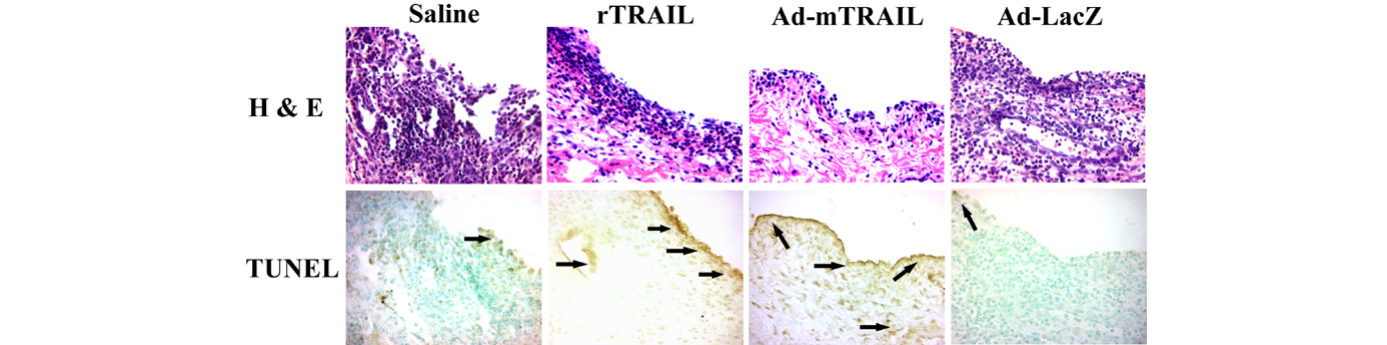
Comparison of the anti-arthritic effects of recombinant TRAIL (rTRAIL) with those of adenoviral-mediated gene transfer of TRAIL. Arthritic rabbit knee joints, induced by intra-articular injection of IL-1β producing cells, were injected intra-articularly with 20 μg of rTRAIL per knee or saline as well as 1 × 10^11 ^particles of adenoviral membrane bound murine TRAIL (Ad-mTRAIL) vectors or adenoviral Lac Z (Ad-LacZ) vectors. The rabbits were sacrificed 48 h post-injection and the joint capsules sectioned to evaluate synovitis. Histological examination of hematoxylin and eosin (H&E) stained synovium sections are shown for saline, rTRAIL, Ad-mTRAIL and Ad-LacZ treated knees. Synovial sections analyzed by TUNEL staining are shown for saline, rTRAIL, Ad-TRAIL and Ad-Lac Z treated joints (200×). Examples of apoptotic cells are indicated by the arrows.

To compare the efficacy of treatment with rTRAIL and Ad-TRAIL gene transfer, rabbit arthritic knee joints were treated intra-articularly with either rTRAIL or Ad-mTRAIL vectors for 48 h. The rabbits were sacrificed and joint capsules sectioned to evaluate synovitis. Histological analysis of H&E stained synovium sections made from both rTRAIL treated and Ad-TRAIL infected joints showed less synovitis than that of saline and Ad-Lac Z controls (Figure 4a–d). Analysis of the sections by TUNEL staining showed extensive apoptosis of synoviocytes from both rTRAIL treated and Ad-mTRAIL treated joints compared to saline and Ad-Lac Z treated joints (Figure 4e–h). There was no significant difference between rTRAIL treated and Ad-mTRAIL treated rabbit arthritic joints, however, suggesting that rTRAIL is as efficient as gene transfer of TRAIL for inducing synovial apoptosis.

## Discussion

TRAIL is a member of the TNF family of ligands, able to induce cell death [18] through association with the death-domain containing receptors, TRAIL-R1 (DR4) and TRAIL-R2 (DR5) [19,20]. TRAIL is able to induce apoptosis of a wide variety of human tumor cells, but generally appears not to affect normal cells. RA synoviocytes exhibit features of partial transformation, and the tumor-like proliferation of synoviocytes in RA thickened joints may result from an imbalance between cell growth and death. To test the possibility that TRAIL might be able to induce apoptosis of arthritic synovium, we previously examined the effects of adenoviral gene transfer of TRAIL. We demonstrated that intra-articular gene transfer of human membrane bound TRAIL into an inflamed rabbit knee resulted in significant apoptosis of the synovium and a reduction in intra-articular leukocytes. Although these previous results suggest that gene transfer of TRAIL could be therapeutic to treat certain pathologies associated with RA, there currently is no appropriate gene transfer system that could be used clinically to deliver TRAIL intra-articularly. Thus, in this report, we have examined the potential therapeutic effects of intra-articular injection of a recombinant chimeric TRAIL protein (rTRAIL). The rTRAIL protein used has been demonstrated to be predominantly trimeric and highly active. We have observed that intra-articular injection of rTRAIL not only results in induction of significant synovial apoptosis, but also reduces joint inflammation as measured by a reduction in the leukocytic infiltrate. These *in vivo *experimental results are consistent with our previous results using an adenoviral vector expressing TRAIL protein. In fact, the histological scores of Ad-TRAIL and rTRAIL treated inflamed rabbit knee joints were similar as well as the percent decrease in the intra-articular white blood cell infiltrate.

In our previous experiments, we found that synovial fibroblasts in culture from different RA patients were partially sensitive to adenoviral mediated TRAIL gene transfer at high multiplicity of infection. It appears as if the apoptotic effect of the over-expression of TRAIL on the synovial fibroblasts from RA patients is mediated predominantly by TRAIL R1 as its expression varied from patient to patient and the extent of apoptosis following TRAIL gene transfer seemed to correlate with its expression intensity (data not shown). This seems to be consistent with the report that TRAIL induces apoptosis by binding to TRAIL R1 and R2. We did not observe any consistent pattern of expression of the TRAIL decoy receptors in the limited number of human synovial fibroblast cultures examined, suggesting that susceptibility to TRAIL is conferred through the level of expression of TRAIL R1 (data not shown). It is important to note, however, that we have been unable to quantify TRAIL receptor levels in rabbit synoviocytes in culture or *in vivo*. It is possible that the proliferating rabbit synovium *in vivo *expresses higher levels of TRAIL receptors. Indeed, the inflammatory process induced by IL-1β expression in the joint may directly or indirectly sensitize the synovial fibroblasts to TRAIL mediated apoptosis.

Our results suggest that the delivery of TRAIL to arthritic joints of rabbits induced apoptosis of synovium to such an extent that significant synovial linings were devoid of intact cells other than acellular debris residing in apparently normal fibrous structures found in the control samples. Clinically, total synovectomy is used for joint fusion operations whereas subtotal synovectomy involving partial removal of hyperplastic synovium, preserving some functions of residual synoviocytes such as secretion of synovial fluid as a lubricant, result in improvement in arthritic scores. The 'molecular synovectomy' using TRAIL could simulate the partial surgical ablation of synovium as well as reduce the leukocytic infiltration.

In addition to inducing apoptosis of the synovium, intra-articular injection of exogenous TRAIL protein reduced the leukocytic infiltrate into the inflamed joints, indicating that TRAIL may inhibit joint inflammation. The mechanism through which TRAIL functions to reduce leukocytosis in inflamed joints is unclear. Interestingly, it appears that it does not cause a reduction in a specific type of leukocyte, but instead appears to reduce the overall leukocytic infiltrate. It is possible that TRAIL reduces the leukocytic infiltrate through the direct induction of apoptosis of intra-articular leukocytes [21]. It is also possible that elimination of the synovium results in a reduction in the intra-cellular pro-inflammatory cytokines such as IL-1, IL-6, IL-8 and TNF. Alternatively, it is possible that induction of extensive apoptosis within the joint space serves to confer a general anti-inflammatory effect, possibly through uptake of apoptotic cells by macrophages and dendritic cells [22]. In this regard, we have observed that all agents able to induce synovial apoptosis in the rabbit knee joints also were able to reduce the extent of the white blood cell infiltrate. Furthermore, the reduction in leukocytosis correlated with the extent of synovial apoptosis. The one exception was with adenoviral gene transfer of FasL, which resulted in synovial apoptosis but also an increase in neutrophil infiltration. It is important to note that multiple mechanisms may be involved in the reduction of the white blood cell infiltrate. It is also important to note that TRAIL-mediated apoptosis of the injected, IL-1β expressing synovial cells used to induce disease also could play a role in the reduction in severity of the inflammatory process.

The partial elimination of the hyperplastic synovium by intra-articular injection of pro-apoptotic agents appears to be therapeutic, but there is concern that the same agents could have adverse effects on the underlying cartilage. However, in our experiments following intra-articular adenoviral mediated TRAIL gene transfer or with rTRAIL, no adverse effects were observed on cartilage metabolism. Thus, at least in the rabbit knee model, intra-articular injection of rTRAIL confers significant therapeutic effects without conferring adverse effects on cartilage.

Taken together, the results with rTRAIL, similar to the results with the adenoviral vector expressing TRAIL, suggest that the intra-articular injection of exogenous TRAIL protein confers both significant apoptotic and anti-inflammatory effects in a rabbit knee model of arthritis. These studies support the further development of therapies based on both local and systemic administration of rTRAIL to treat pathologies associated with RA.

## Conclusions

The results of this study clearly demonstrate that intra-articular injection of the recombinant human TRAIL protein results in induction of synovial apoptosis and reduction of the leukocytic infiltrate in inflamed rabbit knees, similar to the effect of intra-articular adenoviral mediated TRAIL gene transfer. These results suggest that intra-articular injection of rTRAIL could offer therapeutic benefits for the treatment of RA.

## Abbreviations

Ad = adenoviral; Ad-mTRAIL = adenoviral vector expressing membrane bound murine TRAIL protein; AIA: Antigen induced arthritis; GAG = glycosaminoglycan; H&E = hematoxylin and eosin staining; IL = interleukin; LSF = lavage synovial fluid; MTT = 3-(4,5-dimethylthiazol-2-yl)-2,5-diphenyltetrazolium bromide; PBS: Phosphate buffered saline; RA = rheumatoid arthritis; rTRAIL = recombinant TRAIL protein; TNF = tumor necrosis factor; TRAIL = TNF-related apoptosis-inducing ligand; TUNEL = terminal deoxynucleotidyl transferase-mediated dUTP nick end labeling.

## Competing interests

The University of Pittsburgh has patented viral-mediated arthritis gene therapy. The technology has been licensed to Tissuegene, Inc. for which PDR serves as a member of the Scientific Advisory Board. PDR is also on the Scientific Advisory board for Orthogen, GmB. D-WS has a patent on rTRAIL in Korea.

## Authors' contributions

QPY performed the rabbit arthritic knee experiments, injecting rTRAIL protein. In these animal experiments, QPY was assisted by ZM. DWS generated and provided the recombinant chimeric TRAIL protein. PDR conceived of the study and participated in its design and coordination and helped to edit the manuscript. All authors have read and approved the final manuscript.

## Figures and Tables

**Table 1:** Semi-quantification of synovial apoptosis

Group	Synovium	*P* value
Saline (n = 4)	0.625 ± 0.479	
rTRAIL 5 μg (n = 4)	2.500 ± 1.000	0.022
rTRAIL 20 μg (n = 4)	3.000 ± 0.816	0.023

A semi-quantitative scale was used to estimate the degree of TUNEL staining in synovial tissue sections. Investigators blindly reviewed primarily two areas: synovial lining and subsynovial regions. The extent of staining in these areas was scored on a scale of 0 to 4 as follows: 0, negative; 1+, rare positive apoptotic bodies; 2+, scattered clusters of positive apoptotic bodies; 3+, moderate staining; and 4+, extensive staining. Student's *t* test was used for a statistical analysis. rTRAIL, recombinant TRAIL protein.
